# Impact of climate change on groundwater resource in a region with a fast depletion rate: the Mississippi Embayment

**DOI:** 10.2166/wcc.2021.326

**Published:** 2021-09-01

**Authors:** Ying Ouyang, Yongshan Wan, Wei Jin, Theodor D. Leininger, Gary Feng, Yuguo Han

**Affiliations:** USDA Forest Service, Center for Bottomland Hardwoods Research, 775 Stone Blvd., Thompson Hall, Room 309, Mississippi State, MS 39762, USA; Center for Environmental Measurement and Modeling, US EPA, 1 Sabine Island Drive, Gulf Breeze, FL 32561, USA; Bureau of Watershed Management & Modeling, St. Johns River Water Management District, Palatka, FL 32178, USA; USDA Forest Service, Center for Bottomland Hardwoods Research, 432 Stoneville Road, Stoneville, MS 38776, USA; USDA-ARS, Genetic and Sustainable Agricultural Research Unit, 810 Hwy 12 East, Mississippi State, MS 39762, USA; Beijing Forestry University, School of Soil and Water Conservation, Key Laboratory of State Forestry Administration on Soil and Water Conservation, Beijing 100083, China

**Keywords:** climate change, groundwater resource, MERAS model, Mississippi Embayment

## Abstract

Mississippi Embayment (ME) is one of the fastest groundwater depletion regions around the world, while the impacts of climate change on groundwater resources in the region are complex and basically unknown. Using the U.S. Geological Survey’s Mississippi Embayment Regional Aquifer Study (MERAS) model, such a challenge was addressed through the base, wet, and dry simulation scenarios. Over the 137-year simulation period from 1870 to 2007, the cumulative aquifer storage depletions were 1.70 × 10^11^, 1.73 × 10^11^, and 1.67 × 10^11^ m^3^, respectively, for the base, dry, and wet scenarios. As compared with that of the base scenario, the aquifer storage depletions were only 1.76% more for the dry scenario and 1.8% less for the wet scenario. A multiple regression analysis showed that the aquifer storage depletion rate was controlled more by the groundwater pumping and stream leakage rates and less by the groundwater net recharge rate. Groundwater table variation in the forest land was much smaller than in the crop land. Results suggested that groundwater pumping rather than climate change was a key driving force of groundwater depletion in the ME. Our findings provide a useful reference to water resource managers, foresters, and farmers in the ME and around the world when developing their groundwater supply strategies.

## INTRODUCTION

Groundwater depletion due to increasing water demand for agricultural, domestic, and industrial uses is a critical concern worldwide (Gleeson *et al.* 2012; Scanlon *et al.* 2012; Famiglietti 2014; Scanlan 2018). Many parts of the world, including north Africa, Middle East, south and central Asia, north China, north America, and Australia, are now experiencing groundwater resource depletion (Konikow & Kendy 2005; Garduño & Foster 2010; Feng *et al.* 2013; Doll *et al.* 2014; Dalin *et al.* 2017; Ouyang *et al.* 2019). This is also true in the Mississippi Embayment (ME) of USA (Konikow 2013), which encompasses the following eight states (from north to south): Missouri, Illinois, Kentucky, Arkansas, Tennessee, Mississippi, Alabama, and Louisiana. The ME is a key region for crop productions in midsouth USA (Clark & Hart 2009; MSU Extension Service 2014). To maximize crop yields, the crop land area of Mississippi Delta (within the ME) with groundwater irrigation has increased 92% since 1998 and has resulted in a significant depletion of groundwater resources with an average loss of groundwater level greater than 7 m since 1970 (Vories & Evett 2014; YMD 2015; Ouyang *et al.* 2016). It was reported that the ME is one of the fastest groundwater depletion regions in the world (Aeschbach-Hertig & Gleeson 2012).

Climate change is a natural phenomenon, but anthropogenic activities such as fossil fuel burning, industrial pollution, deforestation, and population growth have greatly accelerated the greenhouse gas emissions (Ertürk *et al.* 2014), which have, in turn, resulted in extreme abnormal climate change patterns (IPCC 2012). Climate change over the last several decades is linked to atmospheric water vapor content increase, precipitation pattern shifts, snow cover reduction and ice melt, and surficial hydrological cycle changes (IPCC 2012). More specifically, climate change has made the Northern Hemisphere mid-latitudes wetter, Northern Hemisphere subtropics and tropics drier, and Southern Hemisphere subtropics and deep tropics moister (Zhang *et al.* 2007). IPCC (2012) predicted that precipitation events are very likely to change in intensity, frequency, and location throughout the 21st century, while Tank *et al.* (2009) estimated that temperatures in 2100 are expected to be 1.1–6.4 °C higher than in 1900, accompanied by changing rainfall rate and duration.

Although the impacts of climate change upon surficial water resources are primarily through the long-term changes in air temperature, precipitation, and evapotranspiration, the quantifications of such impacts are complex and may not be directly scaled from changes in temperature and precipitation. In general, groundwater resources are linked to climate change through interactions with rivers, streams, and lakes; sea-level rise and saltwater intrusion; and groundwater evapotranspiration and recharge. Numerous modeling studies investigated climate change impacts on groundwater resources in the past two decades (Doll 2009; Goderniaux *et al.* 2011; Ferguson & Gleenson 2012). Doll (2009) estimated the impacts of climate change on the global vulnerability of renewable groundwater resources using the Water GAP model. She found that the North African rim of the Mediterranean Sea has highest vulnerabilities, whereas the southwestern Africa, northeastern Brazil, and central Andes are the areas of moderate to high vulnerabilities. Goderniaux *et al.* (2011) predicted climate change impacts on groundwater resources in the Geer basin, Belgium, using transient stochastic climatic scenarios. They reported that the climate change impact is stronger than that of natural climate variability by 2085. Ferguson & Gleenson (2012) reported that coastal aquifers are more vulnerable to groundwater extraction than to sea-level rise due to climate change and only aquifers with very low hydraulic gradients may be vulnerable to sea-level rise. Russo & Lall (2017) explored the depletion and response of deep groundwater to climate-induced pumping variability across the USA. They showed that the time period for deep groundwater level response to climate change is less than 1 year. Crosbie *et al.* (2013) projected the potential climate change effects on groundwater recharge in the High Plains Aquifer, TX, USA, using the soil–vegetation–atmosphere transfer model WAVES and global climate model scenarios. Using the dry and wet future climate scenarios, they predicted that the change in groundwater recharge rate is in a range of greater than 50% of the current recharge rate. Ranjan *et al.* (2006) assessed the effects of climate and land use changes on groundwater resources in coastal aquifers in the lower Walawe River basin of southern Sri Lanka through computer modeling. They cited that deforestation increases groundwater recharge in arid areas. Combining climate and land use changes, they showed that when the aridity index is less than 60, the agricultural lands have higher groundwater recharge than other land uses. In contrast, using the HSPF (Hydrological Simulation Program-FOR-TRAN) model, Ouyang *et al.* (2019) recently reported that forest land slightly increases rather than reduces groundwater recharge as compared with that of the agricultural land in the lower Yazoo River watershed, Mississippi, USA.

Clark & Hart (2009) developed the Mississippi Embayment Regional Aquifer Study (MERAS) model to estimate the changes of groundwater flow conditions in the ME from 1870 to 2007. They found that the total flow (sum of inflows or outflows) ranged from about 2.27 million m^3^/d prior to irrigation-development to 68.88 million m^3^/d after irrigation-development near the end of the simulation period in 2006. The pumpage from wells represents the largest outflows, which are offset primarily by inflow from aquifer storage and recharge. However, a thorough search from the literature reveals that no effort has been devoted to assessing the impacts of climate change on groundwater resources in the ME, which is a critical concern from the farmers, foresters, and water resource managers in the region. Although the aforementioned studies have provided useful insights on how climate change affects groundwater resources and the land use affects groundwater recharge, our literature search reveals that very little effort has been devoted to assessing groundwater resource sustainability and temporospatial distribution in crop and forest lands from the faster depletion regions of the USA (e.g., ME and Texas High Plains), which are vitally important for water resource managers to mitigate water availability stress for human use and natural ecosystems as well as to develop adaptive management strategies.

The goal of this study was to assess climate change impacts on groundwater resources in the ME using the MERAS model, which is a site-specific MODFLOW model developed by the U.S. Geological Survey (USGS) (Clark & Hart 2009). MERAS is one of the largest site-specific MODFLOW models in the USA and around the world. The advantages of using the MERAS model for the purpose of this study are as follows: (1) MERAS covers the watersheds of interest in this study with a more than 100-year simulation period, (2) MERAS has been rigorously calibrated and validated by USGS; (3) no other site-specific MODFLOW model like MERAS is available for use in our region; and (4) it is time-saving and cost-effective to adapt the MERAS model rather than to develop a new one. Our specific objectives were to (1) modify the historical groundwater net recharge rates in the MERAS model to reflect the possible future wet and dry climate conditions; (2) import the MERAS model into the ModelMuse simulating system for better pre- and post-processing of the model inputs and outputs; and (3) apply the model to assess the impacts of climate change on groundwater resources in the crop and forest lands as well as the entire ME. Descriptions of the MODFLOW, MERAS, ModelMuse, and simulation scenarios used in this study are given in the section below.

## MATERIALS AND METHODS

### Models descriptions

Developed by USGS, MODFLOW is well known and widely used around the world and is a modular three-dimensional finite-difference groundwater flow simulation model (McDonald & Harbaugh 1988). Since its first release in early 1980, several versions of MODFLOW have been developed. A complete description of the model and its most recent expansion can be found elsewhere (McDonald & Harbaugh 1988; Harbaugh 2005; https://www.usgs.gov/mission-areas/water-resources/science/modflow-and-related-programs?qt-science_center_objects=0#qt-science_center_objects).

**Figure 1 |:**
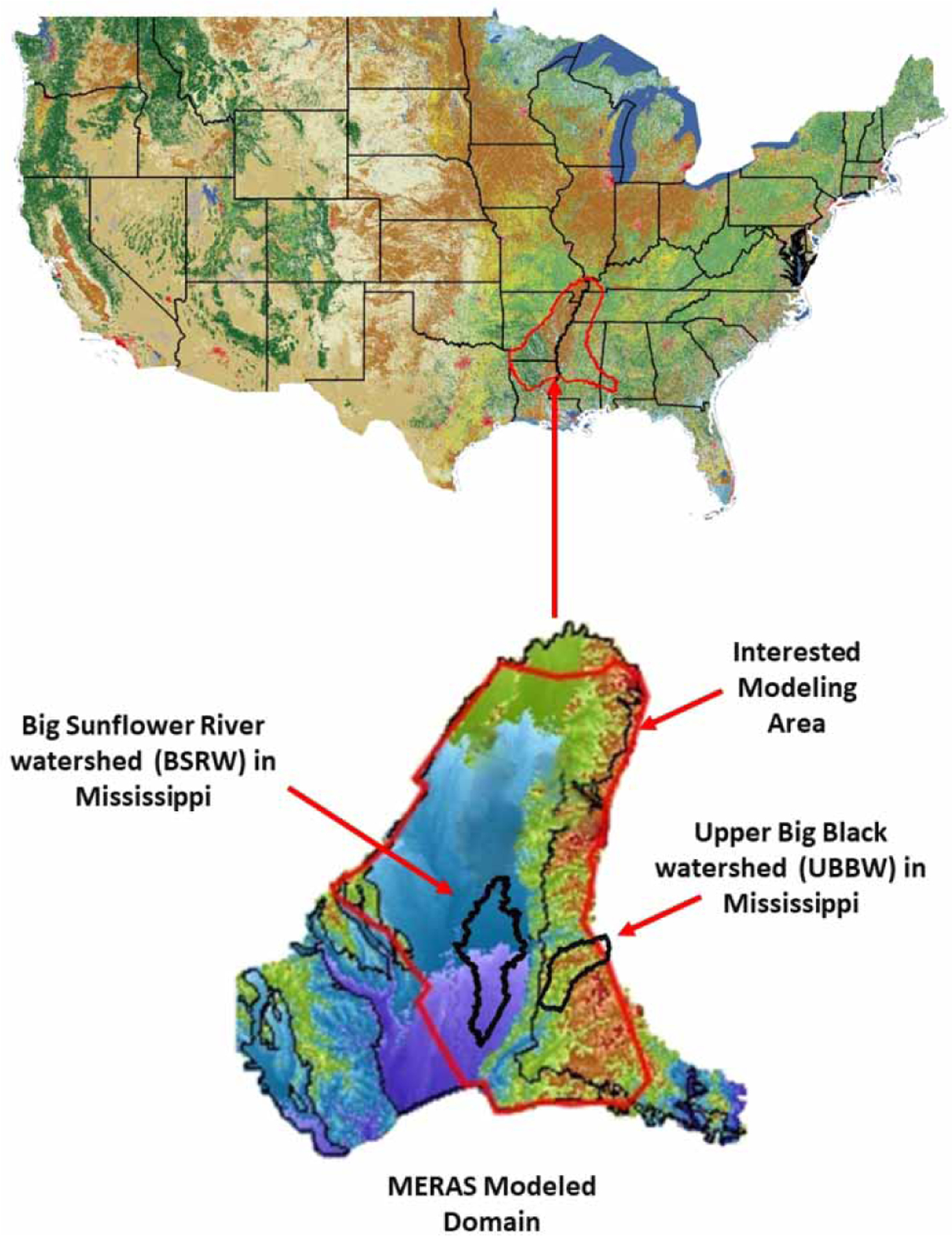
Location of the MERAS modeled domain.

MERAS was developed using MODFLOW-2005 by USGS to assess groundwater resources in the ME (Clark & Hart 2009; Clark *et al.* 2013). The modeling area is about 202,019 km^2^ and includes eight states of the USA (Figure 1). The hydrologic boundaries of the model mainly include areal groundwater net recharge, well pumping, stream routing, and no-flow. The areal groundwater net recharge rate was calculated as a function of precipitation based on topography, soil type, and land use through model calibration. Groundwater pumping is estimated using the Multi-Node Well (MNW) Package in MODFLOW. No-flow boundaries are used for the perimeter of the modeling area, where flow into or out of the model is assumed to be negligible. Initial conditions are obtained using a steady-state stress period prior to 1 January 1870. The model is calibrated and validated prior to its applications. It should be noted that the version of MERAS modified by USGS in 2013 (Clark *et al.* 2013) was used in this study. A detailed description of the boundary and initial conditions is reported in Clark & Hart (2009).

The USGS ModelMuse is a graphical user interface software package that consists of MODFLOW, MODPATH, ZONEBUDGET, PHAST, SUTRA, MT3D, and WellFootprint models and is freely available in the public domain (USGS 2020). ModelMuse is grid independent and stress-period independent. These features provide flexibility for users to freely redefine the spatial and temporal discretizations. ModelMuse is used in this study for better post-processing and graphic display of simulation results.

### Study site

In addition to the entire MERAS modeling area, two watersheds were also selected with one representing a crop land (i.e., the Big Sunflower River watershed or BSRW) and the other representing a forest dominated land (i.e., the Upper Big Black watershed or UBBW). These watersheds are within the MERAS modeled domain (Figure 1). The BSRW (7,666 km^2^) is an intensive crop production area in the alluvial plain with 75.82% of agricultural land and 0.22% of forest land, whereas the UBBW (8,800 km^2^) has less crop production area and situates in the upper hill with 17.05% of agricultural land, 49.19% of forest land, and 15% of shrub/grassland. It should also be noted that there are more than 2,000 groundwater pumping wells in the BSRW as compared with about 20 wells in the UBBW. The average annual precipitation in the BSRW and UBBW is about 1,300 mm and most of the rain falls in winter and spring, with highest rainfall in March and lowest in October. An average annual temperature is 18 °C, with 90 days above 32 °C and 30 days below 0 °C. The climate of the entire ME is moderate with a mean annual precipitation of 1,200 mm in the north to 1,400 mm in the south. Much of the precipitation is lost through evapotranspiration and runoff to the streams (Clark & Hart 2009). The BSRW was selected as the crop land because it is the most important watershed for crop production in the ME, whereas the UBBW was selected as the forest land because it is close to the BSRW with a similar attitudinal range (Figure 3(a)). A similar attitudinal range is necessary for climate change impact comparisons.

### Simulation scenarios

Three simulation scenarios were developed in this study. The first was a base scenario for the agricultural pumping conditions commonly used as well as for the forest conditions that naturally occurred in the ME. The second was the same as the first scenario except that the groundwater net recharge rate was increased by 2%, which represented the wet conditions due to the climate change; whereas the third was the same as the first scenario except that the groundwater net recharge rate was decreased by 2%, which represented the dry conditions due to the climate change. Ouyang *et al.* (2019) recently estimated the groundwater net recharge in the lower Yazoo River watershed, which is located at the south of the BSRW and within the ME. They reported that only about 1.2% of precipitation finally recharges into the groundwater in this area because of the thick clay layer. Based on this research finding, we modified the MERAS model to vary the groundwater net recharge rate by ±2% for our simulation scenarios. Our justifications are that to vary the groundwater net recharge rate by ±2%, the current precipitation rate must increase or decrease by two-fold. Such precipitation events are not very likely to occur in the ME due to the future climate change and are considered as the extreme climate change events. To change the groundwater net recharge by ±2%, the Multiplier File Package (file name ‘meras_trSoils.mlt’) of the MERAS model was modified accordingly. It should be noted that groundwater net recharge in the ME included the processes of precipitation infiltration, stream leakage, and irrigation infiltration return flow to groundwater (Clark & Hart 2009). The groundwater net recharge is the sum of precipitation infiltration, stream leakage, and irrigation infiltration return flow minus the sum of evapotranspiration, surface runoff, and vadose zone soil storage.

It is also worth mentioning here that our simulation scenarios did not include the climate change impact on groundwater pumping rate. In many regions, climate change is expected to induce a change in groundwater pumping rate with an increasing trend under dry conditions and a decreasing trend under wet conditions. However, this may not be the case in the ME. Although there is a high amount of precipitation in this region, only 30% occurs during the crop growing season from May to October (Ouyang *et al.* 2018). The temporal mismatch between annual precipitation and crop water demands is a major reason to pump groundwater for crop irrigation. Our hypothesis is that even under the extreme wet or dry conditions, we do not expect the pumping rate to have a major change because most of the precipitation events do not occur during the growing season and there is no evidence to prove such a precipitation pattern would change in the foreseeable future. Our simulation started on 1 January 1870 and ended on 31 March 2007 for a total simulation period of 137 years with 69 stress periods. It should be noted that the current version of MERAS model only predicts groundwater flow up to 2007.

## RESULTS AND DISCUSSION

### Groundwater table under the base scenario

**Figure 2 |:**
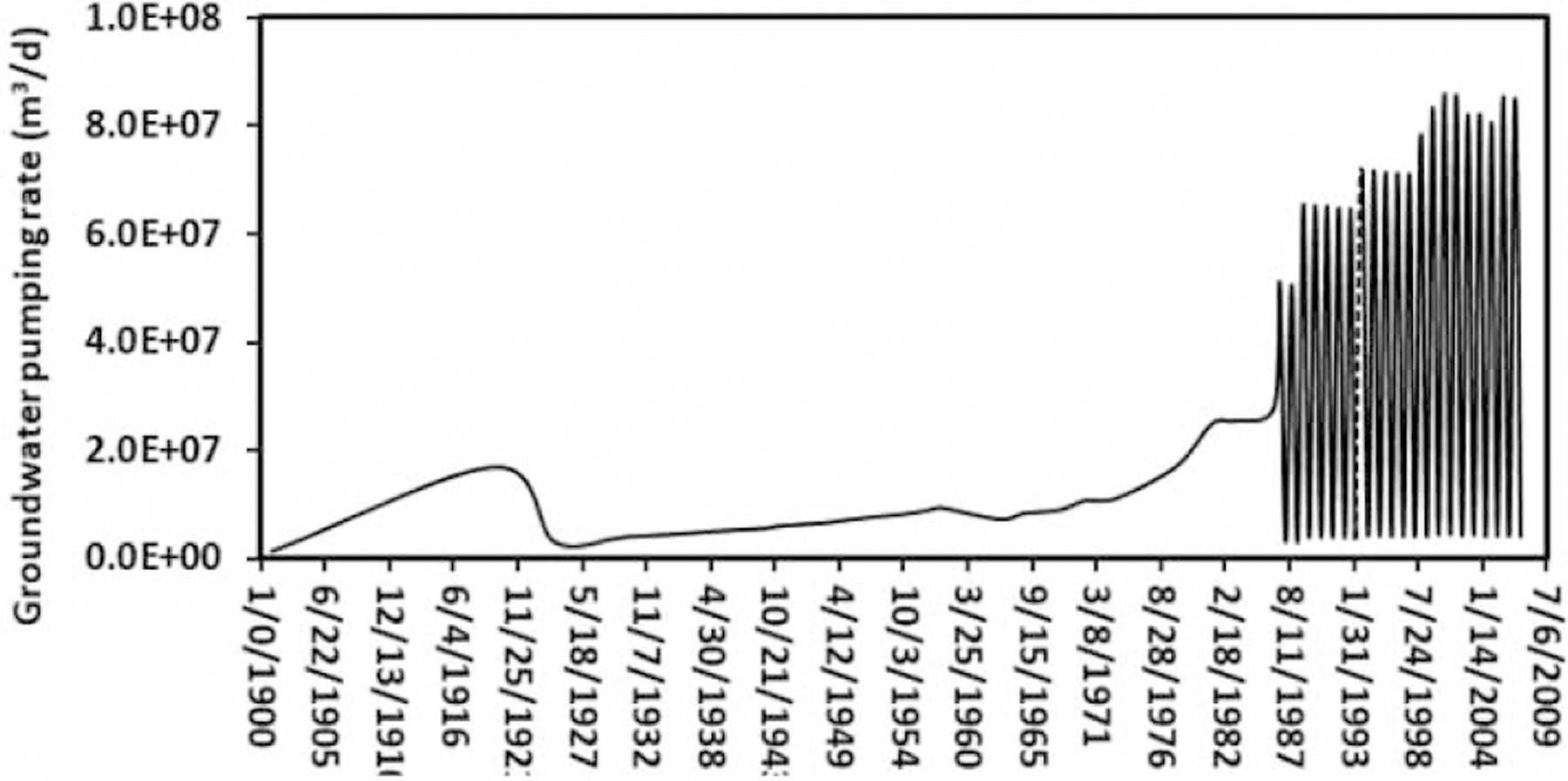
Groundwater pumping rate from 1900 to 2007 used in this study.

It is reported that groundwater table (referenced to the National Geodetic Vertical Datum of 1929 or NGVD 29) in the ME has changed dramatically since 1987 due to the high demand of groundwater pumpage (Figure 2) for crop irrigation (Clark & Hart 2009). Therefore, our focuses on groundwater table were primarily on the simulation results after 1987 (or from 1988).

**Figure 3 |:**
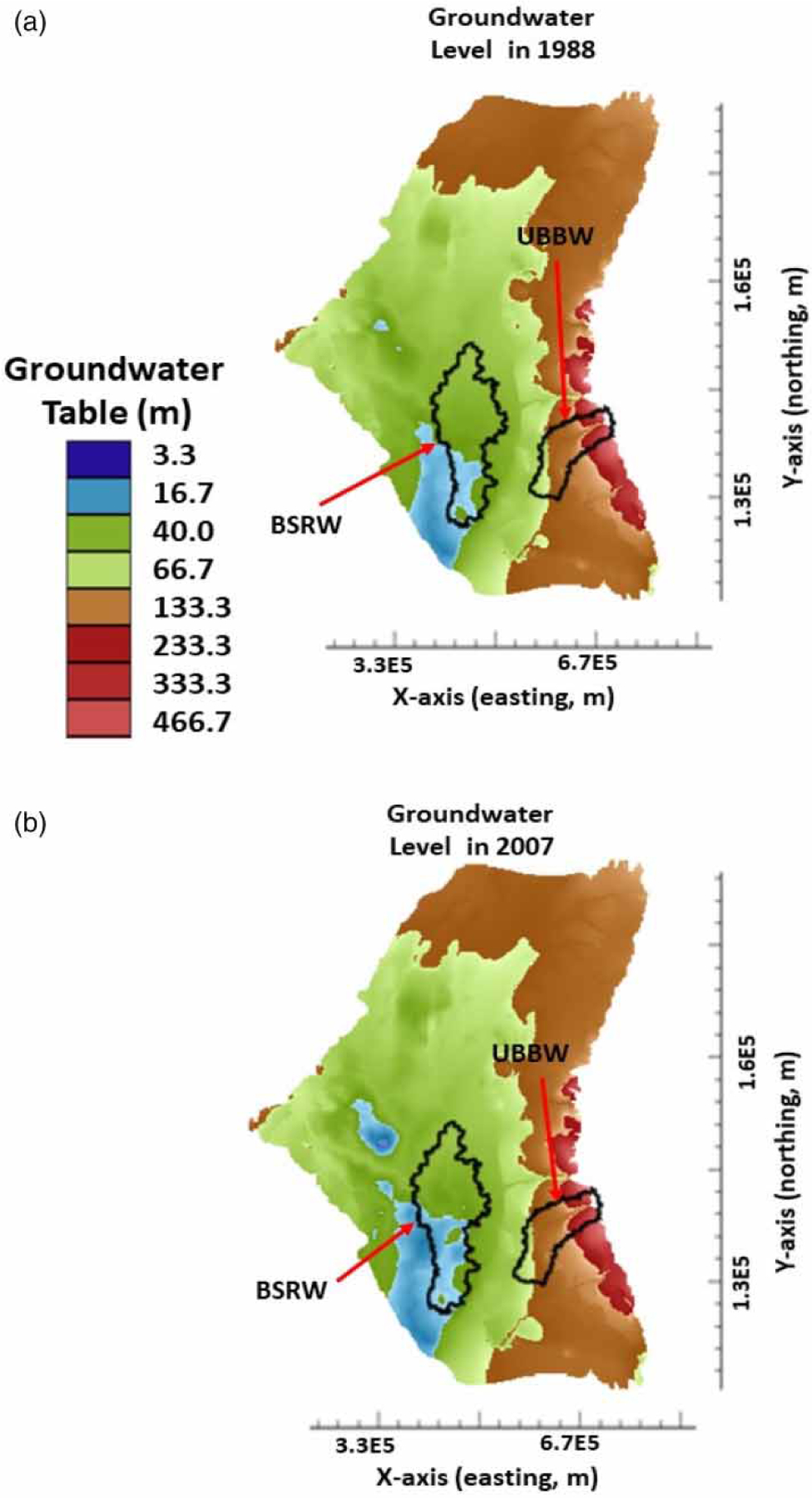
Spatial distributions of groundwater table in 1988 and 2007 for the base simulation scenario.

Spatial distributions of groundwater table in the ME for the base scenario at the simulation years of 1988 and 2007 are given in Figure 3. Only the active modeling area (or area of interest, 1.59 × 10^11^ m^2^) is shown in Figure 3, which encompasses the crop dominated watershed (i.e., BSRW) and the forest dominated watershed (i.e., UBBW). In general, groundwater table was lower in the southwest than in the east and north of the ME. The lowest groundwater table was about 16.7 m in the southwest of the ME, while the highest groundwater table about 466 m in the east of the ME (Figure 3(a)). Over a 20-year simulation period from 1988 (Figure 3(a)) to 2007 (Figure 3(b)), the groundwater table declined for the base scenario. For example, the area was 1.49 × 10^6^ m^2^ in 1988 (Figure 3(a)) with the groundwater table at 16.7 m but was 2.39 × 10^6^ m^2^ (Figure 3(b)) in 2007 with the same groundwater table. The latter had a 60% increase (or more groundwater table decline) in the area than the former.

A similar groundwater depletion trend was found in the area with the groundwater table 66.7 m. That is, the area was 1.59 × 10^7^ m^2^ in 1988 (Figure 3(a)) with the groundwater table at 66.7 m but was 1.74 × 10^7^ m^2^ (Figure 3(b)) in 2007 at the same groundwater table. There was about a 9.4% increase (or more groundwater table decline) in the area at this groundwater table after 20 years from 1988 to 2007. Results demonstrated that groundwater table had declined in the ME and we attributed this decline primarily to the groundwater pumping for crop irrigation. The average groundwater pumping rate during the irrigation period had increased from about 2.5 × 10^8^ m^3^/d in 1988 to 7.6 × 10^8^ m^3^/d in 2007 (Figure 2). This was equivalent to a threefold increase in groundwater pumping rate during the growing season over the 20-year period.

Figure 3(a) further reveals that groundwater table was lower in the agricultural dominated watershed (i.e., BSRW) than in the forest dominated watershed (i.e., UBBW). This occurred because the BSRW is located at the lowland of the Mississippi River Alluvial Valley, while the UBBW is situated at the Mississippi bluff hill. After 20 years from 1988 to 2007, the groundwater table in the BSRW became deeper (Figure 3(b)). More specifically, the area with the groundwater table at 16.7 m in the BSRW increased from 1988 to 2007 (Figure 3(b)). Again, we attributed the increase in the area with the lower groundwater table to the well pumping for crop irrigation.

In contrast, very small changes were observed in the forest dominated watershed (i.e., UBBW) over the 20-year simulation period from 1988 (Figure 3(a)) to 2007 (Figure 3(b)). Most of the area in the UBBW was still within the groundwater table range of 66.7–233.3 m during this period. This occurred because there were only about 20 groundwater pumping wells in the UBBW as compared with more than 2,000 groundwater pumping wells in the BSRW. In other words, the use of groundwater for crop irrigation in the UBBW was minimal. Results further confirmed that groundwater pumping was a major driving force on groundwater table decline for the base scenario.

### Impact of climate change on groundwater table

**Figure 4 |:**
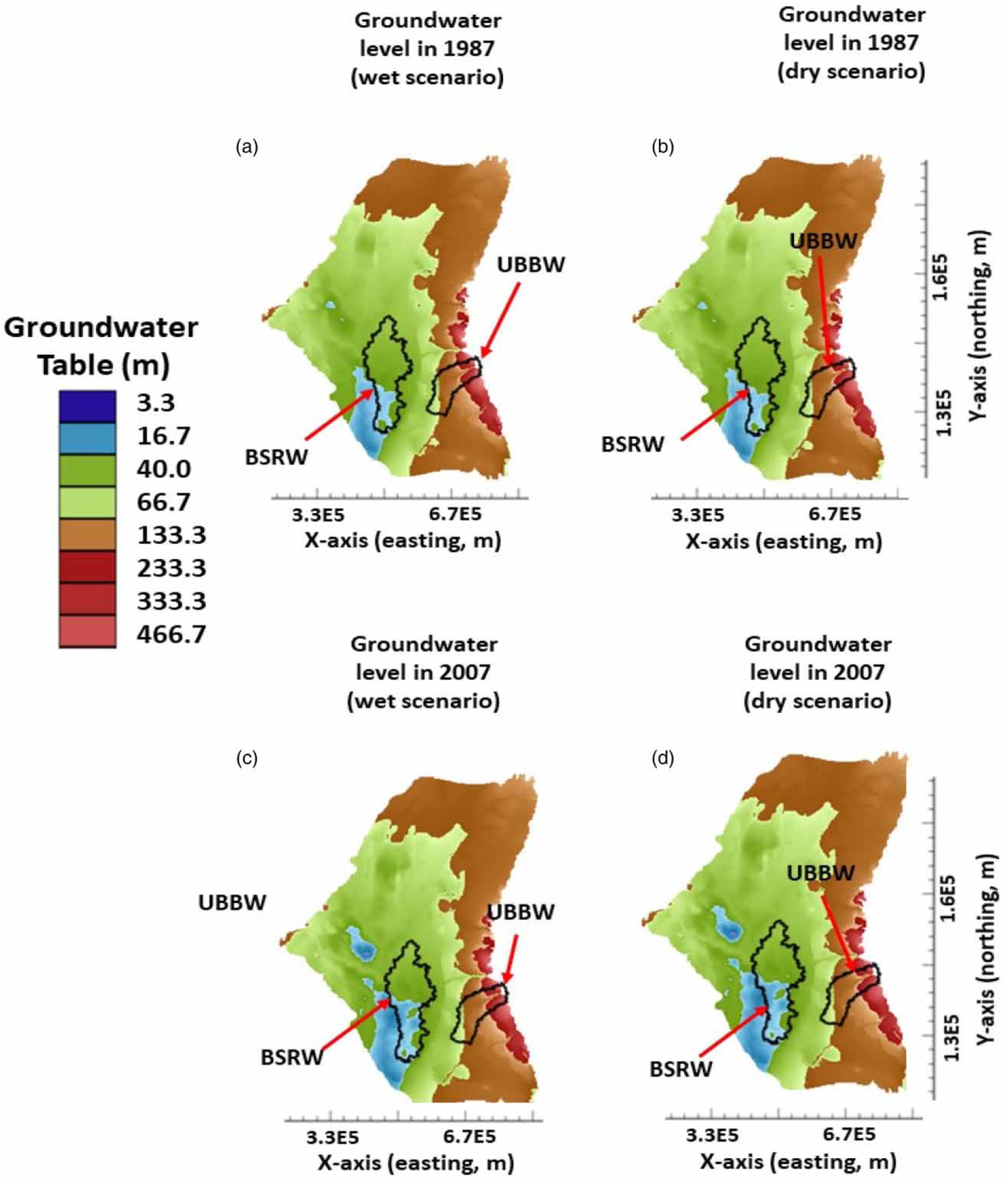
Spatial distributions of groundwater table in 1988 and 2007 for the wet and dry simulation scenarios.

**Figure 5 |:**
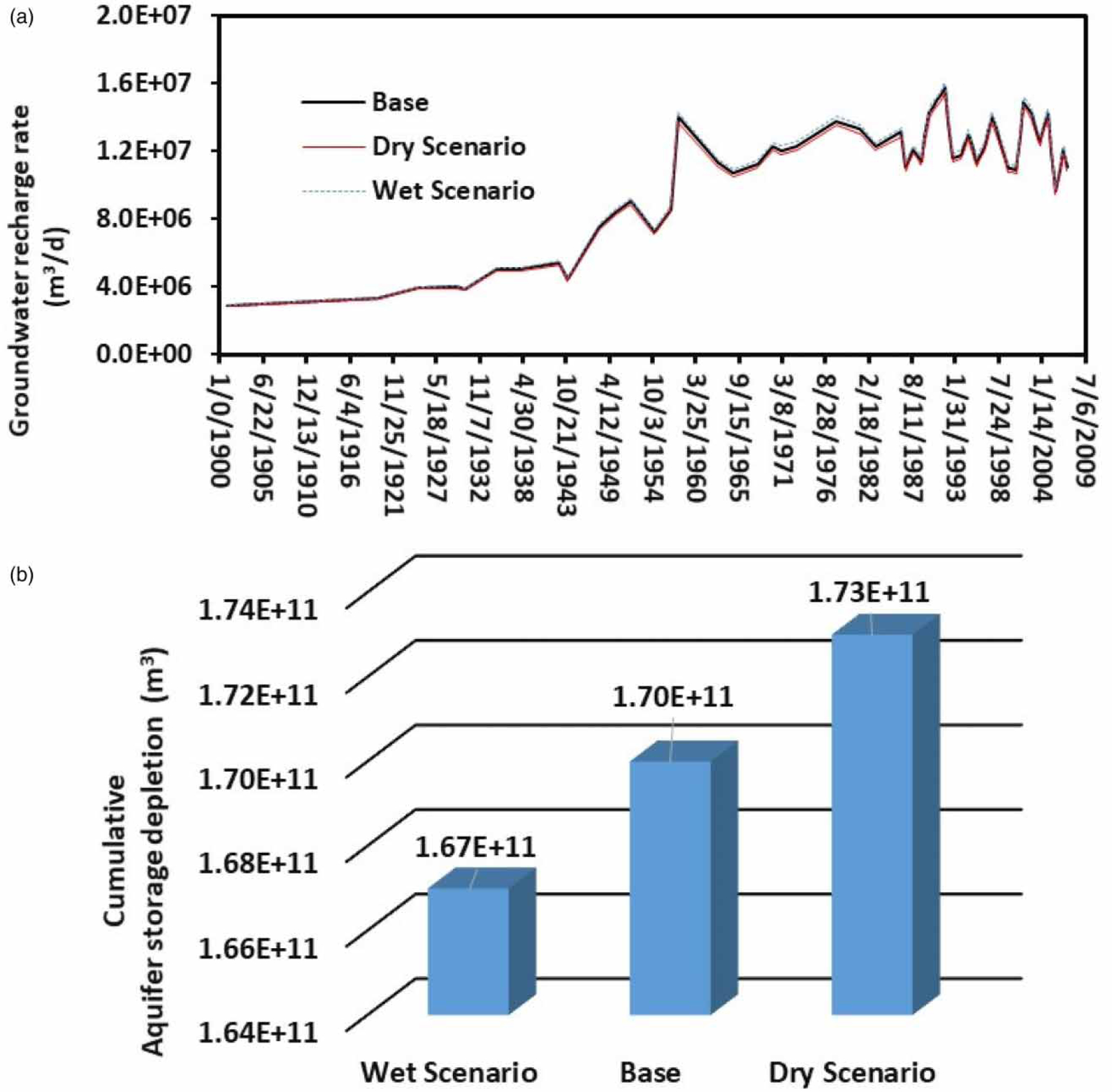
Groundwater net recharge rate (a) and cumulative aquifer storage depletion (b) for the wet, base, and dry simulation scenarios.

Comparisons of spatial distributions of groundwater table in the ME for the three simulation scenarios showed that the impacts of climate change on groundwater resources were somewhat small (Figures 3 and 4). More specifically, the groundwater table distributions in 1988 were almost alike for the base (Figure 3(a)), wet (Figure 4(a)), and dry (Figure 4(b)) scenarios. Similar results were also observed for the groundwater table distributions in 2007 (Figures 3(b), 4(c), and 4(d)). For example, the area with the groundwater table at 16.7 m from 1988 to 2007 increased 59.8% for the base scenario, 51.9% for the wet scenario, and 64.5% for the dry scenario. As compared with that of the base scenario, the wet scenario decreased the area with the groundwater table at 16.7 m by 7.9% (or 59.8–51.9%), whereas a dry scenario increased the area with the groundwater table at the same level by 4.7% (or 64.5–59.8%). Results indicated that the impact of climate change (through groundwater net recharge) on groundwater table distributions in the ME was small. This occurred because there were only slight changes in groundwater recharge rate (±2%) (Figure 5(a)) for the wet and dry simulation scenarios due to climate change. These slight changes in groundwater recharge rate used in this study were legitimate in light of our previous study (Ouyang *et al.* 2019). Ouyang *et al.* (2019) found that only about 1.2% of the precipitation finally recharges into groundwater in a watershed in the ME area and most of the precipitation enter into streams through surface runoff or into the atmosphere through evapotranspiration due to the thick clay layer in most parts of the ME, which prevents precipitation water from infiltrating into groundwater. To have a 2% increase or decrease in groundwater recharge rate, the current precipitation rate might need to increase or decrease twice. Such precipitation events are unlikely to occur in the ME due to future climate change and are considered as the extreme climate change events. Even under such extreme conditions, their impacts on groundwater table distributions were small. This can also be deducted from Figure 5(b). The cumulative aquifer storage depletions at the end of the 137-year simulation were 1.70 × 10^11^, 1.73 × 10^11^, and 1.67 × 10^11^ m^3^, respectively, for the base, dry, and wet scenarios. As compared with that of the base scenario, there was only 1.76% more in aquifer storage depletion under the dry scenario over the 137 years from 1870 to 2007, while there was only 1.8% less in aquifer storage depletion under the wet scenario over the same simulation period. Results further confirmed that climate change did not have discernable impacts on groundwater resources in the ME.

**Figure 6 |:**
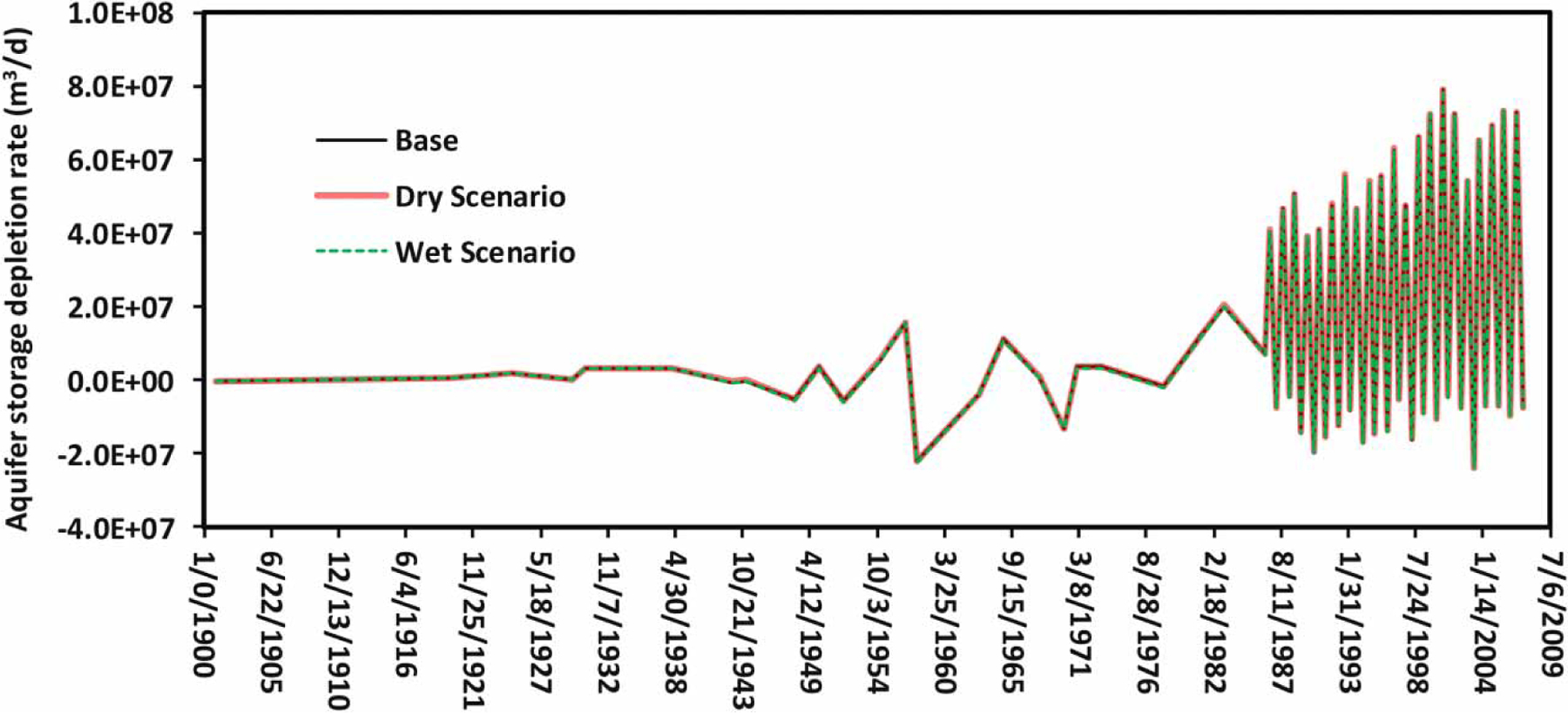
Aquifer storage depletion rates for the base, wet, and dry simulation scenarios from 1900 to 2007.

A time series plot of the simulated aquifer storage depletion rate for the three scenarios over a 107-year simulation period from 1900 to 2007 is given in Figure 6. For all of the three scenarios, the aquifer storage depletion rates were almost constant from 1900 to 1937, then varied with a moderate increasing trend from 1937 to 1986, and finally, fluctuated with an increasing trend from 1987 to 2007. There were clear cuttings in forests and hydrologic channel modifications from 1937 to 1987, which had resulted in groundwater storage variations (Clark & Hart 2009). An increase in aquifer storage depletion occurred from 1987 to 2007 because of the intensive groundwater pumping for crop irrigation (Figure 2) during this period (Clark & Hart 2009; YMD 2015). Figure 6 also demonstrated that the impact of climate change on the aquifer storage depletion rate was negligible because there were very few differences among the three simulation scenarios.

### Impact of subsurface hydrological processes on goundwater storage

Since the impact of climate change on the groundwater resources in the ME was small, it is vitally important to determine the key hydrological process that contributed to groundwater depletion in this region for the purpose of water resource management. Based on our current observations, the areal groundwater net recharge, stream leakage, and groundwater pumping are the three major hydrological processes that interact with groundwater resources in the ME. A multiple linear regression was performed to understand the effects of groundwater pumping, net recharge, and stream leakage on groundwater storage from the base scenario for the entire ME. A strong multiple linear correlation existed between the aquifer storage depletion rate and the pumping, stream leakage, and net recharge rates in the ME as given below:

(1) $$Y=-1.0224x_{1}+2.6438x_{2}+0.0001x_{3}\;\left(R^{2}=0.9869,p<0.001\;\text{for}\;x_{1},x_{2}\;\text{and}\;x_{3}\right)$$

where *Y* is the aquifer storage depletion rate (m^3^/d), *x*_1_ is the pumping rate (m^3^/d), *x*_2_ is the leakage rate (m^3^/d), and *x*_3_ is the recharge rate (m^3^/d). Three distinct patterns were found from the equation: (1) the aquifer storage depletion rate increased as the groundwater pumping rate increased (or more negative); (2) the aquifer storage depletion rate decreased as the stream leakage rate (into the aquifer) increased; and (3) the effect of groundwater net recharge rate on the aquifer storage depletion rate was marginal.

## CONCLUSIONS

Groundwater table in the ME had declined dramatically due to the groundwater pumping for crop irrigation. There was about a 60% increase (or more groundwater depletion) in the area with groundwater table at 16.7 m and about 9.4% increase in the area with the groundwater table at 66.7 m over a 20-year period from 1988 to 2007 for the base scenario. The average groundwater pumping rate during the irrigation period had increased threefold from 1988 to 2007.

Very small changes in groundwater table were observed in the forest dominated watershed (i.e., UBBW) over the 20-year period and occurred because there were about 100 times fewer groundwater pumping wells in the forest dominated watershed than in the agricultural dominated watershed.

Climate change (through the groundwater net recharge) did not have discernable impacts on groundwater table distributions, while groundwater pumping was a key driving force for groundwater table decline in the ME.

A strong multiple linear correlation existed between the aquifer storage depletion rate and the pumping, leakage, and net recharge rates in the ME. Three distinct patterns were found: (1) the aquifer storage depletion rate increased as the groundwater pumping rate increased (or more negative); (2) the aquifer storage depletion rate increased as the stream leakage rate increased; and (3) the effect of groundwater net recharge rate on the aquifer storage depletion rate was marginal.

The major assumption of this study is that the impact of climate change on the groundwater pumping rate was not very significant in the ME. In many regions, climate change is expected to induce a change in groundwater pumping rate with an increasing trend under the dry conditions. Further study is warranted to tackle this issue.

## Data Availability

Data cannot be made publicly available; readers should contact the corresponding author for details.

## HIGHLIGHTS

- One of the world largest groundwater models (i.e., MERAS) was applied.
- Agricultural pumping rather than climate change is a key driving force of groundwater depletion.
- Aquifer storage depletion was controlled by the groundwater pumping and stream leakage.
- Groundwater table variation in the forest land was much smaller than in the crop land.
